# Work-related stress and burnout: Is epigenetic aging the missing link?

**DOI:** 10.1186/s13148-025-01968-z

**Published:** 2025-09-09

**Authors:** Julian Eder, Friederike Sophie David, Sabrina Illius, Nicole Rothe, Magdalena Katharina Wekenborg, Andreas Walther, Marlene Penz, Juulia Jylhävä, Robert Miller, Clemens Kirschbaum, Nina Alexander

**Affiliations:** 1 Chair of Biopsychology, Faculty of Psychology, TUD Dresden University of Technology, Dresden, Germany; 2 Institute of Human Genetics, University of Bonn, School of Medicine & University Hospital Bonn, Bonn, Germany; 3 Department of Psychiatry and Psychotherapy, Philipps University Marburg, Marburg, Germany; 4 Department of Psychology, Faculty of Human Sciences, Medical School Hamburg, Hamburg, Germany; 5 ICAN Institute for Cognitive and Affective Neuroscience, Medical School Hamburg, Hamburg, Germany; 6 Else Kroener Fresenius Center for Digital Health, Faculty of Medicine and University Hospital Carl Gustav Carus, TUD Dresden University of Technology, Dresden, Germany; 7 Psychotherapy and Psychotherapy Research, University of Graz, Graz, Austria; 8 Institute of Psychology, Johannes Kepler University, Linz, Austria; 9 Faculty of Medicine and Health Technology and Gerontology Research Center (GEREC), University of Tampere, Tampere, Finland; 10 Department of Medical Epidemiology and Biostatistics, Karolinska Institutet, Stockholm, Sweden; 11 Chair of Psychological Methods, Psychologische Hochschule Berlin, Berlin, Germany; 12 Center for Mind, Brain and Behavior, Philipps University Marburg, Marburg, Germany

**Keywords:** Burnout, Depression, DNA methylation, Epigenetic clock, Glucocorticoids, Longitudinal, Work-related stress

## Abstract

**Background:**

Work-related stress is a well-established contributor to mental health decline, particularly in the context of burnout, a state of prolonged exhaustion. Epigenetic clocks, which estimate biological age based on DNA methylation (DNAm) patterns, have been proposed as potential biomarkers of chronic stress and its impact on biological aging and health. However, their role in mediating the relationship between work-related stress, physiological stress markers, and burnout remains unclear.

**Methods:**

Here, we analyzed DNAm data from 296 employed individuals (*n*_female_ = 202; *M*_age_ = 45.4; *SD*_age_ = 11.3; range_age_ = 19.5–67.1) from the longitudinally assessed cohort of the Dresden Burnout Study to investigate whether epigenetic aging mediates the relationship between work-related stress (effort–reward imbalance), hair glucocorticoids (cortisol, cortisone), and burnout symptoms. We examined four epigenetic clocks (DNAm Skin&Blood Age, DNAm PhenoAge, DNAm GrimAge, and DNAm GrimAge2) at baseline and follow-up (one year later). Additional mediation analyses were conducted for depressive symptoms to distinguish their potential effects from those specifically associated with burnout symptoms.

**Results:**

As expected, work-related stress at baseline significantly predicted burnout (β = .47, *p* < .001) and depressive symptoms (β = .32, *p* < .001) at follow-up. However, epigenetic aging did not mediate these relationships, neither cross-sectionally (indirect effects of epigenetic age acceleration [EAA]: ß_burnout_ = [−.0008, −.00001]) nor longitudinally (indirect effects of changes in raw clock estimates: ß_burnout_ = [−.002, .007]). Furthermore, work-related stress and hair glucocorticoids were not significantly associated with any epigenetic age markers (all *p* values > .117), and both EAA and changes in epigenetic aging over time were unrelated to burnout or depressive symptoms (all *p* values > .190). Sensitivity analyses adjusting for blood cell composition and technical variance confirmed these findings.

**Conclusions:**

Consequently, our results do not support the hypothesis that epigenetic aging serves as a biological mechanism linking work-related stress or biological stress markers to burnout symptoms. While work-related stress significantly predicts burnout and depressive symptoms, its association does not appear to be driven by epigenetic aging pathways in a low to moderately burdened population. These findings underscore the need for longer follow-up studies to explore alternative biological and psychosocial pathways that shape the long-term consequences of work-related stress on mental health.

**Supplementary Information:**

The online version contains supplementary material available at 10.1186/s13148-025-01968-z.

## Introduction

According to the World Health Organization, work-related stress represents an increasing health burden in Western societies, leading to significant costs in terms of human suffering and economic loss [1–3]. In this context, the effort–reward imbalance (ERI) model provides a conceptual framework suggesting that a lack of reciprocity between personal effort and workplace rewards is a key driver of long-term adverse health outcomes [4]. A critical condition in this regard is burnout—a syndrome characterized by three dimensions, namely emotional and physical exhaustion, negative attitudes toward work, and a diminished sense of professional efficacy, which develops in response to chronic adverse working conditions [5, 6]. Burnout is estimated to affect up to 9 million people in Germany [7], whereas prevalence estimates of burnout vary strongly, as burnout still lacks a validated uniform definition ([8], see also [9]). Besides its strong association with mental disorders [10], burnout constitutes a risk factor for a wide range of physical diseases, like cardiovascular diseases [11]. Moreover, the harmful effects of work-related stress are not limited to mental disorders but also include dysregulation of central stress response systems [12] and various health risks [13, 14] ultimately influencing life expectancy [15].

Despite extensive research, the precise mechanistic pathways linking work-related stress to burnout remain largely unclear [16–18]. A growing body of evidence suggests that chronic psychosocial stress—including work-related stress—may contribute to biological aging processes [19, 20]. Several studies have demonstrated associations between self-reported stress or physiological stress markers and key hallmarks of aging [21], such as oxidative stress [22], telomere shortening [23, 24], and cellular senescence [25, 26]. These findings support the assumption that sustained stress exposure accelerates biological aging, which may in turn increase vulnerability to mental and physical illness.

Within this framework, DNA methylation (DNAm)-based biomarkers, particularly epigenetic clocks, have emerged as promising tools to quantify biological age. While the first generation of epigenetic clocks (e.g., Horvath, see [27, 28]) was designed to accurately predict chronological age, they demonstrated only a relatively limited ability in predicting age-related phenotypes and mortality risk. This led to the development of second-generation clocks (e.g., GrimAge, PhenoAge), which capture more biological variation and were explicitly designed to estimate chronic disease risk and time to death [29, 30]. Interestingly, first- and second-generation epigenetic clocks show only marginal to moderate correlations [31, 32], suggesting that they capture different aspects of the aging process. Epigenetic age acceleration (EAA), defined as having an epigenetic age greater than expected based on one's chronological age, has been linked not only to multiple somatic health risks [33, 34] but also to various stress-related mental health disorders, including post-traumatic stress disorder [35] and depression [36]. However, findings have been inconsistent across studies. Likewise, while some research has identified associations between EAA and different types of environmental adversities [34], other studies have failed to confirm these links [37].

The biological plausibility of stress-related effects on EAA is supported by findings that certain DNAm clocks (e.g., Horvath) contain CpG sites that are particularly responsive to glucocorticoid signaling, a major downstream outcome of hypothalamic–pituitary–adrenal (HPA)-axis activation. Zannas et al. [38] reported substantial dynamic methylation changes at CpG sites relevant to Horvath's epigenetic clock following synthetic glucocorticoid administration. Additionally, longitudinal studies have demonstrated bidirectional associations between EAA and HPA-axis activity**,** where changes in EAA can either precede or follow alterations in cortisol concentrations [39, 40]. Work-related stress, which has been associated with sustained HPA-axis dysregulation [41], may thus contribute to biological wear-and-tear over time.

Despite growing evidence for a potential role of stress-induced EAA in adverse health outcomes, very few studies have explicitly examined the effects of work-related stress. Initial cross-sectional analyses from the UK Understanding Society Study and the Northern Finland Birth Cohort 1966 found that working more than 40 h per week, night shifts, and job insecurity were associated with EAA, while job strain, active work, and white-collar employment were linked to slower epigenetic aging [42, 43]. Drawing on data from the Dresden Burnout Study (DBS), a prospective cohort study with annual multi-level monitoring of an employed population [44] we sought to extend these findings in several ways. First, we employed a longitudinal approach to investigate the mediating role of EAA and changes in epigenetic aging over one year in the association between work-related stress (i.e., ERI) and burnout symptoms. Longitudinal designs are essential for understanding the biological sequelae of work-related stress and its effects on health outcomes, given prior evidence of bidirectional associations between EAA and stress-related disorders [45, 46]. Second, we aimed to complement subjective measures of work-related stress with hair glucocorticoid concentrations, a valid index of HPA-axis activity and a potential driver of burnout symptoms [47] and EAA. Based on previous findings, we hypothesized that EAA would significantly mediate both cross-sectional and longitudinal associations between hypercortisolism and burnout symptoms. Lastly, given the ongoing debate regarding the differentiation between burnout and depression [48], we aimed to investigate whether stress-induced changes in EAA specifically relate to burnout or whether they generalize to depressive symptoms. By combining longitudinal assessments of psychological symptoms, glucocorticoid activity, and epigenetic aging, the present study provides a unique opportunity to investigate whether EAA and changes in epigenetic aging over one year partially mediates the impact of chronic stress on mental health outcomes—a pathway previously proposed for depression [49], but not yet examined in the context of burnout.

## Methods

### Study design

This study was part of the DBS, a longitudinal cohort study designed to explore the burnout syndrome in depth by examining both biological and psychosocial factors over time [44]. The primary goal of the study is to identify potential biomarkers and gain insights into the long-term progression and impact of burnout in a German-speaking population aged 18–68 years. Participants are recruited through age-stratified random sampling via population registries, media platforms, and invitation letters sent to private households. Each year, they complete online questionnaires covering demographic data, burnout-related indicators, workplace conditions, and health-related factors. Additionally, a subset of participants undergoes laboratory-based biomarker assessments, which include endocrine, immunological, and epigenetic analyses using blood and hair samples. The study was approved by the TUD Dresden University of Technology ethics commission (EK236062014), conducted in accordance with the Declaration of Helsinki, with all participants providing written informed consent and receiving monetary compensation for their participation.

### Participants & procedure

Out of the 9911 individuals who registered for the annual online DBS assessments, approximately 3,800 residents of Saxony were invited for on-site assessment—including biomarker collection—between 2015 and 2022. For the present project, 300 participants were selected for a nested cohort study focusing on epigenetic markers to investigate the mechanistic pathways linking work-related stress to burnout. To this end, inclusion criteria required complete data on work-related stressors at baseline and burnout symptoms from at least two DBS assessment waves (baseline [T0] and follow-up [T1], one year apart). Additionally, participants needed to provide a hair sample for steroid analysis and a blood sample for DNA methylation analysis. Only participants who completed the online assessment on stress and health-related factors within ± 4 months before or after the on-site (baseline) biomarker assessment were included in the study. Individuals who reported being pregnant or who had given birth within the previous three months were excluded. A comprehensive description of the assessment waves is provided by Penz et al. [44] as well as at https://osf.io/rjktd and https://osf.io/mz8nu. For a visual summary, please refer to Fig. S1 (see Additional File 1), which was created using Mermaid (https://www.mermaidchart.com). The present study was preregistered on July 25, 2024, at the Open Science Framework (https://osf.io/rjktd).

### Assessment of burnout and depressive symptoms

Burnout symptoms were assessed using a German translation of the Maslach Burnout Inventory—General Survey (MBI-GS), designed to evaluate job-related symptoms across various professions [50]. Construct validation supports both a three-factor model, comprising the subscales exhaustion (EX), cynicism (CY), and reduced professional efficacy (rPE), as well as a one-factor model with 16 items [51]. Sample items are “I feel emotionally drained from my work,” “In my opinion, I am good at my job,” and “I doubt the significance of my work” [50]. Each item is rated on a 7-point Likert scale (0 = never; 6 = daily) [52]. In the current study, the time frame for assessment was set within the past 12 months. Mean values of each subscale were weighted as a total score, following the approach of Kalimo et al. [53], yielding a possible range between 0 and 6. The overall MBI-GS score was calculated based on the formula proposed by Kalimo et al. [53]: 0.4 × EX + 0.3 × CY + 0.3 × rPE**.** High internal consistencies were found for all subscales at T0 (EX: *α* = 0.90, CY: *α* = 0.87, rPE: *α* = 0.82) and T1 (EX: *α* = 0.91, CY: *α* = 0.90, rPE: *α* = 0.85) as well as for a total MBI-GS score (T0: MBI-GS: *α* = 0.91; T1: MBI-GS: *α* = 0.92). Absolute agreement between T0 and T1 MBI-GS scores revealed a good intraclass correlation coefficient, ICC[1,3] of 0.82, 95% CI [0.78, 0.85], F(295,295) = 10.17, *p* < 0.001 [54].

Depressive symptoms were assessed via an online questionnaire using the German version of the Patient Health Questionnaire-9 (PHQ-9) [55–57] one week prior to the annual assessment. The PHQ-9 captures depressive symptoms associated with major depressive episodes, based on Diagnostic and Statistical Manual of Mental Disorders (DSM)-IV and DSM-5 criteria [58, 59], covering the past two weeks. Sample items refer to the corresponding depressive symptoms, e.g., “little interest or pleasure in doing things,” “feeling down, depressed, or hopeless” [55]. Each of the nine items is rated on a 4-point Likert scale (0 = not at all; 3 = nearly every day), with the sum score indicating depression severity. Internal consistency, as measured by Cronbach’s α, was 0.86 at T0 and 0.84 at T1. Absolute agreement between T0 and T1 PHQ-9 scores showed moderate-to-good test–retest reliability, ICC[1,3] = 0.76, 95% CI [0.70, 0.80], F(293,293) = 7.24, *p* < 0.001. Some participants showed missing values for depressive symptoms.

### Assessment of effort–reward imbalance

Subjective work-related stress was assessed using the short version of the ERI questionnaire [60]. It comprises three subscales: effort, reward, and overcommitment, comprising 16 items rated on a 4-point Likert scale (1 = strongly agree; 4 = strongly disagree). A sample item for the effort scale is “I have constant time pressure due to a heavy work load,” and a sample item for the reward scale is “I receive the respect I deserve from my superior or a respective relevant person” [60]. The ERI questionnaire demonstrates good factorial validity and satisfactory reliability and is widely used in epidemiological research [61]. The effort–reward imbalance ratio, ranging from 0.25 to 4, was calculated using the effort (three items) and reward (seven items) subscales, along with an item correction factor [60, 62]. Scores > 1 indicate a higher reported effort in work for each perceived reward.

### Hair glucocorticoid analysis

Hair glucocorticoids (cortisol and cortisone) were assessed as biological markers of long-term stress, reflecting cumulative cortisol secretion over time [63]. To obtain long-term integrated hair cortisol (hairF) and hair cortisone (hairE) concentrations, three 3 cm hair strands were cut as close as possible to the scalp from the posterior vertex, representing cortisol accumulation over the past three months [64]. Sample preprocessing and biochemical quantification were conducted using liquid chromatography-tandem mass spectrometry (LC–MS/MS), following standard protocols [65, 66]. Analyses were performed by Dresden LabService GmbH (Tatzberg 47, 01307, Dresden, Germany). The intra- and inter-assay coefficients of variation for cortisol and cortisone were less than 8.8% and 8.5%, respectively, with lower detection limits of 0.09 pg/mg for cortisol and 0.07 pg/mg for cortisone [65]. Additionally, participants completed a standardized hair protocol assessing the number of hair washes per week and hair treatments (e.g., coloration, tinting, permanent wave), which are considered potential confounders of hair glucocorticoid levels [67]. Hair steroid concentrations were log-transformed to account for non-normal distribution and strong skewness.

Due to insufficient hair length, data of *n* = 25 subjects could not be collected. Moreover, cortisol and cortisone samples differ due to the number of non-detectable values in cortisol (*n* = 9). Extreme outliers in hair cortisol and cortisone (≥ 3 IQR from the median of the log-transformed outcomes) were excluded.

### DNAm profiling and quality control

EDTA whole blood samples from both assessment waves (T0 and T1) were sent to Life & Brain GmbH, Bonn, for DNA extraction and methylation profiling using the Infinium MethylationEPIC v1.0 BeadChip (Illumina, San Diego, CA, USA). For each individual, the T0 and T1 samples were plated on the same Illumina EPIC array to reduce technical noise. Quality control (QC) was performed using the minfi [68] and ewastools [69] R packages. Samples were excluded if the mean log median intensity in both the methylated and unmethylated channels was below 10.5 (see Fig. S2A, Additional File 1), if the call rate was below 98% (see Fig. S2B, Additional File 1), or if there was a mismatch between data-derived and phenotypic sex (see Fig. S2C, Additional File 1). Additionally, samples were removed if they failed any of the Illumina BeadArray control metrics (*n* = 3, see Fig. S3, Additional File 1) or if the average log-odds score for belonging to the outlier component across all SNP probes was above −4 (*n* = 1). Following data normalization using the “normal-exponential convolution using out-of-band probes” (Noob) approach [70], probes were excluded if the call rate was below 98%, if they contained polymorphic or cross-hybridizing binding sites [71], if they were non-CpG sites, or if they were located on non-autosomal chromosomes. After completing quality control and preprocessing, 596 samples (T0, *n* = 297; T1: *n* = 299) and 768,482 probes remained available for epigenetic clock calculation.

### Epigenetic clock calculation

Epigenetic age estimates at T0 and T1 were obtained using four epigenetic clocks. These included the first-generation “Skin & Blood Clock,” originally trained to predict chronological age (DNAm Skin&Blood Age [28], and three second-generation clocks designed to reflect biological age: DNAm PhenoAge [29], DNAm GrimAge [30], and DNAm GrimAge2 [72]). DNAm Skin&Blood Age and DNAm PhenoAge estimates were calculated in R using the methylclock package, while the calculation of DNAm GrimAge and DNAm GrimAge2 was based on R scripts and regression weights provided by the authors of the DNAm GrimAge model. The latter is equivalent to the implementation in the publicly available DNA Methylation Age Calculator webtool (https://dnamage.clockfoundation.org). In our sample, 385 out of 391 CpGs from the Skin & Blood Clock were available, as well as 509 out of 513 CpGs from the DNAm PhenoAge model. Additionally, 827 out of 1030 CpGs were available for both versions of DNAm GrimAge. EAA was determined by calculating the residuals of epigenetic age estimates regressed on chronological age (see Fig. S4, Additional File 1). For longitudinal analyses, change scores (i.e., Delta_EA) were computed by subtracting baseline epigenetic aging scores from those at follow-up (i.e., Δ-DNAm Skin&Blood Age = DNAm Skin&Blood Age at T1 − DNAm Skin&Blood Age at T0; Δ-DNAm PhenoAge = DNAm PhenoAge at T1 − DNAm PhenoAge at T0; Δ-DNAm GrimAge = DNAm GrimAge at T1 − DNAm GrimAge at T0; and Δ-DNAm GrimAge2 = DNAm GrimAge2 at T1 − DNAm GrimAge2 at T0).

### Statistical analyses

We conducted mediation analyses based on linear regression models to test our hypotheses that epigenetic aging significantly mediates both cross-sectional (i.e., EAA) and longitudinal (i.e., Delta_EA) associations between work-related stress, hypercortisolism, and burnout symptoms. These analyses were performed using PROCESS [73] with all calculations conducted in R (v4.4.1) and RStudio (v2024.09.0 + 375; [74]). Estimation uncertainty was addressed by including bootstrapping (*R* = 1000) and robust standard errors (HC4). The alpha level was set at 0.05. To identify potential confounders, mediators, and collider biases [75], we constructed directed acyclic graphs (DAGs) based on theoretical considerations using dagitty.net [76]. The resulting DAGs are presented in Fig. S5 (see Additional File 1).

As a first step, we included all minimal sufficient adjustment variables as confounders to estimate the total effect. These variables, selected based on the research question, included sex, the exact time difference between the T0 and T1 assessment waves (in days), the time difference between the online assessment of stress and health-related factors and the on-site biomarker assessment (in days), the number of hair washes, hair treatments, and two education dummy variables [77]. Sex was included as a covariate in all models involving DNAm GrimAge and DNAm GrimAge2 to account for its role both as a potential confounder and as a component of the composite clock outcome [30, 72].

In the second step, we included either EAA (cross-sectionally) or Delta_EA (longitudinally) to estimate direct and indirect effects.

In the third step, we conducted sensitivity analyses by incorporating additional confounders relevant to methylation data, including estimated cell composition [78] and Illumina control probe principal components (ctrl-probe PCs) to adjust for technical biases [79]. We included only two ctrl-probe PCs, which accounted for 87.6% of variance, as PC3 was highly correlated and PC4 weakly correlated with sex (see Fig. S6A–6D, Additional File 1). PC5 was excluded as it explained less than 2% of additional variance. Additionally, we included five of the six estimated cell types—CD8 + T cells, CD4 + T cells, natural killer (NK) cells, monocytes, and B cells—to mitigate multicollinearity [80], as granulocytes exhibited the highest variance inflation factor. For the longitudinal sensitivity analyses and the necessary adjustment of Delta_EA, we extracted residual scores from the model *epigenetic_aging_sensitivity* ~ *ctrl-probe PCs* + *estimated-cell-compounds* for both baseline and follow-up measurements. Here, we did not adjust for EAA or baseline epigenetic aging values, as doing so in observational studies can introduce collider bias [81].

## Results

Demographic and health-related characteristics of the study participants are summarized in Table 1. At T0 and T1, the final sample consisted of 296 participants, with a balanced gender distribution (68.2% female). The participants had a mean age of approximately 45.4 years (*SD* = 11.3) at T0, with ages ranging from 19.5 to 67.1 years. The ERI scores reveal an average value of 1.15 (*SD* = 0.45) at T0 for the overall sample, indicating that a significant portion of participants may experience work-related stress due to a perceived mismatch between the effort they put into their work and the rewards they receive in return. Mean scores of burnout symptomatology (*M*_T0_ = 2.04; *SD*_T0_ = 1.04; *M*_T1_ = 2.06; *SD*_T1_ = 1.06) in the overall sample indicate a population which exhibits symptoms almost weekly or a few times a month [53]. Mean sum scores of depression severity (*M*_T0_ = 7.18; *SD*_T0_ = 4.68; *M*_T1_ = 6.64; *SD*_T1_ = 4.34) can be categorized as minimal to moderate in the overall sample [55].

**Table 1:** Sample Characteristics

	Baseline (T0) (*n* = 296)		Follow-up (T1) (*n* = 296)	
Variables	*M* (*SD*)/*n (%)*	*Range*	*M* (*SD*)/*n (%)*	Range
*Sociodemographic data*				
Gender (female, male, diverse*), *female* (*%*)	202 (68.2)		202 (68.2)	
Age in years *M* (*SD*)	45.4 (11.3)	19.5–67.1	46.4 (11.3)	20.2–68.1
*Graduation (%)*				
Secondary school & middle school (%)	61 (20.6)			
Vocational diploma (%)	28 (9.5)			
High-school diploma (%)	207 (69.9)			
*Health-related data*				
BMI in kg/m^2^, *M* (*SD*)	25.47 (4.66)	15.06–42.72	25.83 (4.78)	16.96–42.20
< 18.5^a^, *n* (%)	3 (1.0)		4 (1.4)	
18.5–24.99^b^, *n* (%)	153 (51.7)		145 (49.0)	
25–29.99^c^, *n* (%)	91 (30.7)		98 (33.1)	
≥ 30^d^, *n* (%)	49 (16.6)		49 (16.6)	
Alcohol consumption, ≥ 4-times per week, *n* (%) ^e, f^	47 (15.9)		43 (14.5)	
*AHRR* (cg05575921) hypomethylation,* M* (*SD*)	2.73 (0.78)	− 0.26–4.15	2.72 (0.79)	− 0.46–4.23
Chronic diseases (last 3 months), *n* (%) ^g,h^	68 (23.0)		43 (14.5)	
*Mental health-related data*				
Current severity of depressive symptoms (PHQ-9), Sum score, *M* (*SD*)	7.18 (4.68)	0—23	6.64 (4.34)	0—20
Categorical, n (%)^i^	294 (99.3)		296 (100.0)	
Minimal, 0–4^j^, *n* (%)	111 (37.5)		112 (37.8)	
Mild, 5–9^j^, *n* (%)	90 (30.4)		109 (36.8)	
Moderate, 10–14^j^, *n* (%)	77 (26.0)		62 (20.9)	
Moderately severe, 15–19^j^, *n* (%)	13 (4.4)		10 (3.4)	
Severe, 20–27^j^, *n* (%)	3 (1.0)		3 (1.0)	
Burnout symptomatology (MBI-GS), *M* (*SD*)	2.04 (1.04)	0.00–5.30	2.06 (1.06)	0.00–4.83
Exhaustion, *M* (*SD*)	2.58 (1.41)	0.00–6.00	2.54 (1.42)	0.00–5.80
Cynicism, *M* (*SD*)	1.88 (1.40)	0.00–6.00	1.94 (1.44)	0.00–4.67
Reduced professional efficacy, *M* (*SD*)	1.48 (0.90)	0.00–5.00	1.53 (0.96)	0.00–5.80
Effort–Reward Imbalance (ERI), *M* (*SD*)	1.15 (0.45)	0.25–3.11	–	–
*Biological markers*				
Hair cortisol (pg/mg)*, M* (*SD*)^k,^	8.90 (8.33)	0.20–50.07	–	–
Hair cortisone (pg/mg)*, M* (*SD*)^l^	22.94 (19.50)	0.84–145.98	–	–
DNAm Skin&Blood Age	45.41 (12.39)	16.9–71.12	46.55 (12.27)	17.90–77.02
DNAm PhenoAge	36.97 (12.36)	6.7–67.54	38.55 (11.88)	7.74–74.24
DNAm GrimAge	46.65 (9.63)	26.13–67.99	47.59 (9.61)	25.40–69.34
DNAm GrimAge2	52.61 (9.38)	32.43–72.84	53.45 (9.29)	31.40–75.50
DNAm Skin&Blood Age – EAA	− 0.02 (2.75)	− 7.46–15.13	0.03 (2.96)	− 7.02–19.18
DNAm PhenoAge–EAA	− 0.29 (5.14)	− 14.11–22.39	0.30 (5.11)	− 11.34–30.13
DNAm GrimAge–EAA	− 0.05 (3.40)	− 7.58–13.60	0.07 (3.36)	− 6.65–17.02
DNAm GrimAge2 – EAA	− 0.02 (3.82)	− 8.25–13.47	0.05 (3.76)	− 7.90–17.54

*Note.* Sociodemographic, health-related and mental health-related data are described for T_0_ and T_1_. *M*: Mean, *SD*: Standard Deviation, BMI: Body Mass Index, AHRR: Aryl-Hydrocarbon Receptor Repressor, PHQ-9: Patient Health Questionnaire-9, MBI-GS: Maslach Burnout Inventory—General Survey, DNAm: DNA Methylation. * no subject reported “diverse” as gender category. BMI is grouped into ^a^ underweight; ^b^ normal weight; ^c^ overweight; and ^d^ obesity. ^e^ *n* = 15 missing. ^f^ *n* = 10 missing. *AHRR* (cg05575921) hypomethylation serves as a proxy for smoking. ^g^ *n* = 4 missing. ^h^ *n* = 3 missing. PHQ-9 scores are grouped in terms of depression severity according to Kroenke et al. [55] from minimal to severe. ^i^ *n* = 2 missing. ^j^ indicates the respective range of points for depression severity. Overall MBI-GS score is calculated according to Kalimo et al. [53] using the following formula: 0.4 × exhaustion + 0.3 × cynicism + 0.3 × reduced professional efficacy. ERI is calculated using the effort and reward subscales, along with an item correction factor [60, 62]. ^k^ *n*_cortisol-T0_ = 263. ^l^ *n*_cortisone-T0_ = 272. EAA: Epigenetic Age Acceleration

### Temporal correlations of EAAs and raw epigenetic clock estimates

Pearson correlations were used to assess the temporal associations of both raw clock and EEA estimates between T0 and T1 in our *n* = 296 sample. Raw clock estimates were nearly perfectly correlated over time: DNAm Skin&Blood Age (*r* = 0.99, *p* < 0.001), DNAm PhenoAge (*r* = 0.95, *p* < 0.001), DNAm GrimAge (*r* = 0.99, *p* < 0.001), and DNAm GrimAge2 (*r* = 0.98, *p* < 0.001). EAA measures showed high correlations over time (see Fig. S4B): DNAm Skin&Blood Age EAA (*r* = 0.85, *p* < 0.001), GrimAge EAA (*r* = 0.91, *p* < 0.001), and GrimAge2 EAA (*r* = 0.87, *p* < 0.001), with DNAm PhenoAge EAA exhibiting somewhat lower temporal association (*r* = 0.72, *p* < 0.001).

### Mediating role of EAA in the relationship between work-related stress and burnout symptoms (cross-sectional analysis)

Based on the sample size of *n* = 296 and the available data on work-related stress within ± 4 months of collection of biological samples, the final sample size for analyses with ERI as predictor comprised *n* = 248 participants. As expected, work-related stress, measured by the ERI ratio, was significantly associated with burnout (total and direct effect with all four EAA markers as mediators: β = 0.50, *p* < 0.001, 95% BCI [0.91, 1.42]) and depressive symptoms (β = 0.33, *p* < 0.001, 95% BCI [2.23, 4.61]) at T0 in the cross-sectional models. However, no significant associations were observed between work-related stress and any of the four EAA markers (all *p* values > 0.394), nor between these EAA markers and burnout (all *p* values > 0.725) or depressive symptoms (all *p* values > 0.251) at T0. Consequently, our hypotheses regarding the mediating role of EAA in the relationship between the ERI ratio and burnout (indirect effects: ß = [−0.0008, −0.00001]) or depressive symptoms (indirect effects: ß = [0.00005; 0.004]) were not supported in the cross-sectional analysis. All path coefficients are presented in Table 2 for burnout symptoms, while results for depressive symptoms can be found in Table S1 (see Additional File 1).

**Table 2:** Mediating Role of EAA Regarding Work-related Stress or Hair Glucocorticoids and Burnout Symptoms

	**Outcome Regression Fit**				**Mediation Paths, ß**				**Indirect Effects**	
	***F ***^***HC4***^	***df***	***p***	**R**^**2**^	***a***	***b***	***c***	***c ‘***	**ß**	**95% BootCI**
*Model: ERI T0* →* Burnout symptoms T0* *Confounders: sex, education dummy 1, education dummy 2, time difference ERI completion and baseline assessment*										
DNAm Skin&Blood Age	15.92	6, 241	<.001	.27	.02	−.01	.50***	.50***	−.0001	[−.01,.01]
DNAm PhenoAge	16.24	6, 241	<.001	.27	.06	−.01	.50***	.50***	−.0008	[−.01,.01]
DNAm GrimAge	16.15	6, 241	<.001	.27	.04	−.02	.50***	.50***	−.0007	[−.01,.01]
DNAm GrimAge2	16.28	6, 241	<.001	.27	.001	−.02	.50***	.50***	−.00001	[−.01,.01]
*Model: HairF T0* → *Burnout symptoms T0* *Confounders: sex, education dummy 1, education dummy 2, hair treatment, hair washes*										
DNAm Skin&Blood Age	0.22	7, 237	.980	.01	.03	−.02	−.001	.001	−.0006	[−.01,.01]
DNAm PhenoAge	0.23	7, 237	.979	.01	.08	−.01	−.001	−.0001	−.001	[−.02,.01]
DNAm GrimAge	0.23	7, 237	.979	.01	.05	−.006	−.001	−.0009	−.0003	[−.01,.01]
DNAm GrimAge2	0.25	7, 237	.973	.01	.10	−.02	−.001	.0007	−.002	[−.02,.01]
*Model: HairE T0* →*Burnout symptoms T0* *Confounders: sex, education dummy 1, education dummy 2, hair treatment, hair washes*										
DNAm Skin&Blood Age	0.21	7, 244	.983	.01	.09	−.02	−.03	−.03	−.002	[−.02,.01]
DNAm PhenoAge	0.21	7, 244	.982	.01	.05	−.01	−.03	−.03	−.0005	[−.01,.01]
DNAm GrimAge	0.22	7, 244	.982	.01	.08	−.001	−.03	−.03	−.0001	[−.01,.01]
DNAm GrimAge2	0.23	7, 244	.978	.01	.11	−.02	−.03	−.03	−.002	[−.02,.01]

EAA: Epigenetic Age Acceleration. ERI: Effort–Reward Imbalance. DNAm: DNA Methylation. Reference education dummy 1: secondary school & middle school. Reference education dummy 2: vocational school. HairF = Hair cortisol concentration. HairE = hair cortisone concentration. Model sample size: T0 ERI: *n* = 248; T0 Cortisol: *n* = 245; and T0 Cortisone: *n* = 252. *** *p* <.001

### Mediating role of EAA in the relationship between hair glucocorticoids and burnout symptoms (cross-sectional analysis)

Based on the sample size of *n* = 296 and the available self-reported data on hair treatment and hair washes, the final sample sizes for hair glucocorticoids as biological stress markers were *n* = 245 for cortisol and *n* = 252 for cortisone. In the cross-sectional analysis, no significant associations were observed between hair glucocorticoids and burnout symptoms (*p*_hairF_ = 0.986; *p*_hairE_ = 0.727) or depressive symptoms (*p*_hairF_ = 0.761; *p*_hairE_ = 0.759) at T0. Regarding our main hypotheses, no associations were found between hair cortisol (all *p* values > 0.145) or hair cortisone (all *p* values > 0.117) concentrations and any of the EAA markers investigated, based on the cross-sectional minimal sufficient adjustment models (which included hair washes per week and hair treatment as additional confounders). Consequently, we found no evidence supporting EAA as a mediator in the relationship between hair glucocorticoids and burnout symptoms at baseline (indirect effects: ß = [−0.002, −0.0001]; see Table 2 for detailed path coefficients). A similar pattern of associations was observed for depressive symptoms (indirect effects: ß = [−0.002, 0.006]; see Table S1, Additional File 1, for detailed path coefficients).

### Mediating role of Delta_EA in the relationship between work-related stress and burnout symptoms (longitudinal analysis)

As expected, work-related stress, measured by the ERI ratio, prospectively predicted burnout (total and direct effect with all four EAA markers as mediators: β = [0.46; 0.47], *p* < 0.001, 95% BCI [0.84, 1.37]) and depressive symptoms (β = [0.31; 0.32], *p* < 0.001, 95% BCI [1.91, 4.14]) at T1 in the longitudinal models. However, work-related stress at T0 did not predict longitudinal changes in any of the first- and second-generation epigenetic age markers investigated (all *p* values > 0.118). Similarly, Delta_EA markers over the one-year interval were not associated with burnout (all *p* values > 0.190) or depressive symptoms (all *p* values > 0.375) at T1. Consequently, our hypotheses regarding the mediating role of Delta_EA in the relationship between work-related stress and burnout or depressive symptoms could not be supported in our longitudinal analysis (indirect effects: ß_burnout_ = [−0.002, 0.007]; ß_depressive symptoms_ = [−0.0001, 0.005]). All path coefficients are detailed in Table 3 for burnout symptoms, while results for depressive symptoms are provided in Table S2 (see Additional File 1).

**Table 3:** Mediating Role of Delta_EA Regarding Work-related Stress or Hair Glucocorticoids and Burnout Symptoms

	Outcome Regression Fit				Mediation Paths, ß				Indirect Effects	
	*F *^*HC4*^	*df*	*p*	R^2^	*a*	*b*	*c*	*c ‘*	ß	95% BootCI
*Model ERI T0 *→ *Burnout symptoms T1* *Confounders: sex, education dummy 1, education dummy 2, time difference ERI completion and baseline assessment, time difference T1 − T0*										
Δ-DNAm Skin&Blood Age	11.72	7, 240	<.001	.23	.03	.07	.47***	.47***	.002	[−.01,.02]
Δ-DNAm PhenoAge	12.04	7, 240	<.001	.23	−.08	−.08	.47***	.46***	.007	[−.005,.02]
Δ-DNAm GrimAge	11.94	7, 240	<.001	.23	−.10	.02	.47***	.47***	−.002	[−.02,.01]
Δ-DNAm GrimAge2	11.78	7, 240	<.001	.23	−.08	−.03	.47***	.47***	.002	[−.01,.02]
*Model: HairF T0 *→* Burnout symptoms T1* *Confounders: sex, education dummy 1, education dummy 2, hair treatment, hair washes, time difference T1* − *T0*										
Δ-DNAm Skin&Blood Age	0.13	8, 236	.998	.01	−.06	.01	.01	.01	−.0007	[−.01,.01]
Δ-DNAm PhenoAge	0.39	8, 236	.928	.02	.01	−.11	.01	.01	−.0001	[−.02,.01]
Δ-DNAm GrimAge	0.35	8, 236	.943	.02	−.07	−.09	.006	−.001	.007	[−.01,.03]
Δ-DNAm GrimAge2	0.34	8, 236	.922	.02	−.07	−.09	.006	−.0003	.006	[−.01,.03]
*Model: HairE T0 *→* Burnout symptoms T1* *Confounders: sex, education dummy 1, education dummy 2, hair treatment, hair washes, time difference T1 − T0*										
Δ-DNAm Skin&Blood Age	0.19	8, 243	.993	.01	−.10	.01	−.03	−.03	−.001	[−.02,.01]
Δ-DNAm PhenoAge	0.42	8, 243	.908	.02	.01	−.10	−.03	−.03	−.001	[−.02,.01]
Δ-DNAm GrimAge	0.41	8, 243	.913	.02	−.09	−.09	−.03	−.04	.008	[−.01,.03]
Δ-DNAm GrimAge2	0.43	8, 243	.903	.02	−.07	−.09	−.03	−.04	.006	[−.01,.03]

Delta_EA: Change score of raw clock estimate at T1 − raw clock estimate at T0. ERI: Effort–Reward Imbalance. DNAm: DNA Methylation. Reference education dummy 1: secondary school & middle school. Reference education dummy 2: vocational school. Δ: Change in epigenetic aging between T0 and T1. HairF = Hair cortisol concentration. HairE = hair cortisone concentration. Model sample size: T0 ERI: *n* = 248; T0 Cortisol: *n* = 245; T0 Cortisone: *n* = 252. *** *p* <.001

### Mediating role of Delta_EA in the relationship between hair glucocorticoids and burnout symptoms (longitudinal analysis)

In our longitudinal analysis, hair glucocorticoid levels at T0 did not predict burnout (*p*_hairF_ = 0.939; *p*_hairE_ = .666) or depressive (*p*_hairF_ = 0.355; *p*_hairE_ = 0.982) symptoms at T1. Contrary to our expectations, hair cortisol and hair cortisone concentrations at T0 were not associated with changes in any of the four epigenetic age markers (all *p* values for hairF > 0.329; all *p* values for hairE > 0.168) examined in the longitudinal minimal sufficient adjustment models. Ultimately, we found no evidence supporting the mediating role of Delta_EA in the relationship between hair glucocorticoid levels at baseline and burnout symptoms (indirect effects: ß = [−0.001, 0.008]) at follow-up. For a detailed overview of path coefficients, see Table 3 for burnout symptoms, while results for depressive symptoms (indirect effects: ß = [−0.002, 0.007]) are available in Table S2 (see Additional File 1).

### Sensitivity analysis

Sensitivity analyses incorporating surrogate measures of cell mixture distribution [78] and two Illumina control probe principal components did not yield any significant findings regarding the mediating role of EAA or Delta_EA (see Table S3 and Table S4, Additional File 1). Aside from the significant effect of work-related stress on burnout and depressive symptoms, the only considerable predictor of burnout symptoms was Δ-DNAm Skin&Blood Age (β = 0.11, *p* = 0.044, 95% BCI [0.00; 0.09]). These findings are overall consistent with previous analyses, reinforcing the robustness of our results.

Given the lack of significant findings and considerations of multiplicity, we did not conduct further sensitivity analyses using models that included additional lifestyle and health factors—such as BMI, alcohol consumption, AHRR hypomethylation at cg05575921 as a proxy for smoking [82], and medical illness [77] which could potentially influence the associations between work-related stress, mental health, and EAA.

## Discussion

This study examined both cross-sectional and longitudinal mediating effects of epigenetic aging in the relationship between work-related stress, biological stress markers, and burnout symptoms, with a comparative focus on depressive symptoms. Contrary to our expectations, we found no evidence that EAA (cross-sectionally) or Delta_EA (longitudinally) plays a mediating role in these associations. Additionally, neither work-related stress nor hair glucocorticoid levels were significantly linked to epigenetic aging markers. While previous research has suggested that environmental adversity can accelerate biological aging through DNA methylation changes [83], our findings do not support this mechanism in the context of work-related stress and burnout. Instead, our results reaffirm the strong link between work-related stress and adverse mental health outcomes [84, 85]. The ERI model, which conceptualizes work stress as a mismatch between effort and reward, has been consistently associated with increased burnout [86, 87] and depression [88, 89] emphasizing the need for workplace interventions that reduce stress and promote mental well-being.

The lack of evidence linking work-related stress to EAA generally aligns with previous research findings. The few studies investigating this relationship have primarily relied on cross-sectional designs, limiting their ability to evaluate causality. In an initial study, Freni-Sterrantino, Fiorito, d’Errico, Robinson, et al. [42] examined the association between a broad range of work characteristics, including ERI, and five biomarkers of epigenetic aging in the Northern Finland Birth Cohort 1966 Study. Among the numerous associations tested, only few work stress indicators were significantly linked to EAA, particularly after adjusting for potential confounders (see also [90]). Overall, their findings revealed an inconsistent pattern of associations: While specific markers of EAA were positively associated with longer working hours, they unexpectedly also showed negative associations with higher effort. In a second cross-sectional study, where similar analyses were conducted using data from the UK Understanding Society study, job insecurity and night work were found to predict EAA [43]. However, given the large number of associations tested, the evidence for robust relationships remains limited, and the findings do not generalize across different aging biomarkers (see also [90]). In accordance with our findings, no significant associations between ERI ratio and any of the EAA biomarkers were observed in the Northern Finland Birth Cohort 1966 Study, which was the only one to investigate this specific relationship. This suggests that despite its well-documented impact on mortality and morbidity, ERI may not play a substantial role in advancing epigenetic clock metrics. It is thus possible that specific EAA markers are more sensitive to other environmental stressors, such as maternal prenatal stress [91], childhood trauma ([92, 93], but also see [94), neighborhood deprivation [95], or socioeconomic status [96, 97]. Likewise, albeit speculatively, the investigated tissue type blood, which only serves as a proxy for aging in brain and other nervous tissue, could have failed to adequately reveal these associations (see [98]). Another possibility for the missing longitudinal link is that the timeframe of our study was insufficient to detect significant epigenetic alterations, which can be assumed from the high temporal stability of the epigenetic aging markers. Epigenetic modifications often develop over prolonged periods, particularly in response to chronic stressors [38, 99]. Given that our follow-up period was approximately one year, it is possible that epigenetic changes related to work stressors take longer to manifest or require repeated exposure over a more extended period of time. Still the development of strong stress symptoms was shown to affect the pace of epigenetic aging over a similar period of time [100]. This notion is supported by research demonstrating that cumulative lifetime stress, rather than short-term stress exposure, is a stronger predictor of EAA [101]. Future studies with longer follow-up intervals may be necessary to capture the full extent of EAA following work-related stress.

Notably, the null findings regarding work-related stress in our study are also reflected at the biological level, as no significant associations were found between hair glucocorticoid levels and epigenetic aging markers. This was unexpected, given that a substantial number (*n* = 85) of epigenetic clock CpG sites (*n* = 353 CpG sites based on Horvath [27]) are located within glucocorticoid response elements, rendering their methylation levels responsive to cortisol stimulation [40, 102]. Indeed, significant methylation changes at these CpG sites have been observed as early as three hours after oral administration of synthetic glucocorticoids (1.5 mg of dexamethasone, DEX); however, these changes did not affect DNA methylation-predicted aging [38]. Furthermore, it remains unclear whether DEX-induced methylation changes are merely transient and might not be reflected in long-term glucocorticoid concentrations. While cumulative hair glucocorticoid levels have been understudied in the context of EAA, research on HPA-axis activity has yielded mixed results. One study linked EAA to higher diurnal cortisol production in adolescent girls [39], whereas another found no association with HPA-axis feedback regulation via the dexamethasone suppression test [102]. A long-term study on women with childhood sexual abuse histories found that midlife EAA predicts lower peak cortisol levels and earlier cortisol decline, suggesting that epigenetic aging may shape or amplify HPA-axis function over time [40]. Thus, a potential relationship between HPA-axis activity and EAA is likely bidirectional, influenced by sample characteristics, measurement methods, and temporal context.

Contrary to our hypothesis, we did not find significant associations between EAA and burnout or depressive symptoms in our low to moderately burdened sample. While no prior study has explicitly examined EAA in relation to burnout, our findings partly diverge from previous research on depression. While some studies have linked depression to EAA (e.g., [49, 103–105]), others have reported decelerated aging [37] or no significant difference in aging compared to individuals without depression (e.g., [106, 107]). Consistent with our data, longitudinal studies have also found that baseline EAA does not predict future depressive symptoms [108, 109], nor does depression at baseline predict later EAA [110]. The heterogeneity of these findings may stem from differences in analytical methods across studies [77], variations in sample characteristics, depression severity and duration, and the specific epigenetic clocks used to assess biological aging. Additionally**,** genetic [111] and environmental factors such as early-life adversity [103] may interact with depression to influence epigenetic aging trajectories.

### Strength and limitations

This study has several notable strengths. A key strength is its longitudinal design, which enables the examination of temporal changes in epigenetic aging and stress-related mental health outcomes**.** Despite increasing interest in the relationship between work-related stress, epigenetic signatures, and burnout [112, 113], longitudinal investigations remain scarce**.** Additionally, the use of both subjective (ERI ratio) and objective (hair glucocorticoid) stress markers, reported to be only weekly associated with each other [12], enhances the robustness of our findings. The inclusion of multiple epigenetic clocks further enables a nuanced investigation of different biological aging processes. However, several limitations should be acknowledged. First, the one-year follow-up period may have been insufficient to capture long-term epigenetic changes associated with chronic stress. Future studies should extend the observation period to assess whether stress-related epigenetic alterations emerge over multiple years. Second, our sample primarily consisted of a working population with moderate stress exposure, limiting generalizability to populations experiencing severe or chronic stress. Future studies should examine whether stronger epigenetic effects emerge in high-risk groups, such as healthcare workers or individuals with high occupational demands. Third, the specific blood cell types affected by stress-related epigenetic modifications remain unknown. Some leukocytes, such as neutrophils, have short half-lives and may have been replaced by the time of follow-up, potentially masking earlier methylation changes [114, 115].

#### Conclusion and future directions

In summary, this study does not support the hypothesis that EAA mediates the link between work-related stress, hair glucocorticoids, and burnout symptoms in a low to moderately burdened sample. While work-related stress remains a robust predictor of burnout and depressive symptoms, its effects do not appear to be driven by epigenetic aging mechanisms within the timeframe examined. These findings underscore the need to explore alternative biological pathways that may underlie the long-term effects of work-related stress on mental health such as inflammation [116–118] or metabolic dysregulation [119]. Interestingly, EAA and inflammatory markers are largely independent indicators of biological aging that, when combined, may enhance the prediction of stress-related morbidities [120]. Another promising mediator is vagal flexibility, operationalized as vmHRV (vagally-mediated heart rate variability), which has been associated with stress and shown to predict burnout symptom trajectories [121–123]. Future research should focus on longer follow-up periods, integrating multiple biological stress markers, and examining potential moderating factors such as genetic predisposition [124], lifestyle behaviors [96], and stress resilience [125] mechanisms. A more comprehensive understanding of the complex interplay between the occupational environment, epigenetic aging, and mental health will be crucial for developing targeted interventions to mitigate the long-term health consequences of work-related stress.

## Supplementary Information

Additional file 1.

## Data Availability

The anonymized data and code will be made available upon reasonable request.

## Abbreviations

ERI: Effort-reward imbalance
DNAm: DNA methylation
EAA: Epigenetic age acceleration
HPA: Hypothalamic-pituitary-adrenal
DBS: Dresden burnout study
T0: Baseline
T1: Follow-up
MBI-GS: Maslach burnout inventory – general survey
EX: Exhaustion
CY: Cynicism
rPE: Reduced professional efficacy
ICC: Intraclass correlation coefficient
PHQ-9: Patient health questionnaire-9
DSM: Diagnostic and statistical manual of mental disorders
hairF: Hair cortisol
hairE: Hair cortisone
LC–MS/MS: Liquid chromatography-tandem mass spectrometry
QC: Quality control
Noob: Normal-exponential convolution using out-of-band probes
Delta_EA: Change score (raw clock estimate at T1 − raw clock estimate at T0)
Δ-DNAm Skin&Blood Age: DNAm Skin&Blood Age at T1 − DNAm Skin&Blood Age at T0
Δ-DNAm PhenoAge: DNAm PhenoAge at T1 − DNAm PhenoAge at T0
Δ-DNAm GrimAge: DNAm GrimAge at T1 − DNAm GrimAge at T0
Δ-DNAm GrimAge2: DNAm GrimAge2 at T1 − DNAm GrimAge2 at T0
DAGs: Directed acyclic graphs
Ctrl-probe PCs: Illumina control probe principal components
NK cells: Natural killer cells
AHRR: Aryl-hydrocarbon receptor repressor
BMI: Body mass index
BCI: Bootstrap confidence interval
DEX: Dexamethasone
vmHRV: Vagally-mediated heart rate variability
