# Supplementary material for: Work-related stress and burnout: Is epigenetic aging the missing link?
Source: Clin Epigenetics. 2025 Sep 9;17:148. doi: 10.1186/s13148-025-01968-z (PMC12418647; doi:10.1186/s13148-025-01968-z)
Supplement: Supplementary file 1 — Additional file 1. [file 13148_2025_1968_MOESM1_ESM.docx]

**Work-Related Stress and Burnout: Is Epigenetic Aging the Missing Link?**

Julian Eder^a*^, Friederike Sophie David^b,c^, Sabrina Illius^d,e^, Nicole Rothe^a^, Magdalena Katharina Wekenborg^a,f^, Andreas Walther^g^, Marlene Penz^h^, Juulia Jylhävä^i,j^, Robert Miller^a,k^, Clemens Kirschbaum^a^ & Nina Alexander^c,l*^

*^a^Chair of Biopsychology****,*** *Faculty of Psychology****, TUD Dresden University of Technology,*** *Dresden, Germany*

*^b^Institute of Human Genetics, University of Bonn, School of Medicine & University Hospital Bonn, Bonn, Germany*

*^c^Department of Psychiatry and Psychotherapy, Philipps University Marburg, Marburg, Germany*

*^d^Department of Psychology, Faculty of Human Sciences, Medical School Hamburg, Hamburg, Germany*

*^e^ICAN Institute for Cognitive and Affective Neuroscience, Medical School Hamburg, Hamburg, Germany*

*^f^Else Kroener Fresenius Center for Digital Health, Faculty of Medicine and University Hospital Carl Gustav Carus, TUD Dresden University of Technology, Dresden, Germany*

*^g^Psychotherapy and Psychotherapy Research, University of Graz, Graz, Austria*

*^h^Institute of Psychology, Johannes Kepler University, Linz, Austria*

*^i^Faculty of Medicine and Health Technology and Gerontology Research Center (GEREC), University of Tampere, Tampere, Finland*

*^j^Department of Medical Epidemiology and Biostatistics, Karolinska Institutet, Stockholm, Sweden*

*^k^Chair of Psychological Methods, Psychologische Hochschule Berlin, Berlin, Germany*

*^l^Center for Mind, Brain and Behavior, Philipps University Marburg, Marburg, Germany*

*^*^Corresponding authors*

julian.eder@tu-dresden.de

fdavid@uni-bonn.de

sabrina.illius@medicalschool-hamburg.de

nicole.rothe@tu-dresden.de

magdalena.wekenborg@tu-dresden.de

a.walther@psychologie.uzh.ch

marlene.penz@jku.at

juulia.jylhava@ki.se

r.miller@phb.de

clemens.kirschbaum@tu-dresden.de

[nina.alexander@uni-marburg.de](mailto:nina.alexander@uni-marburg.de)

**Additional File 1**


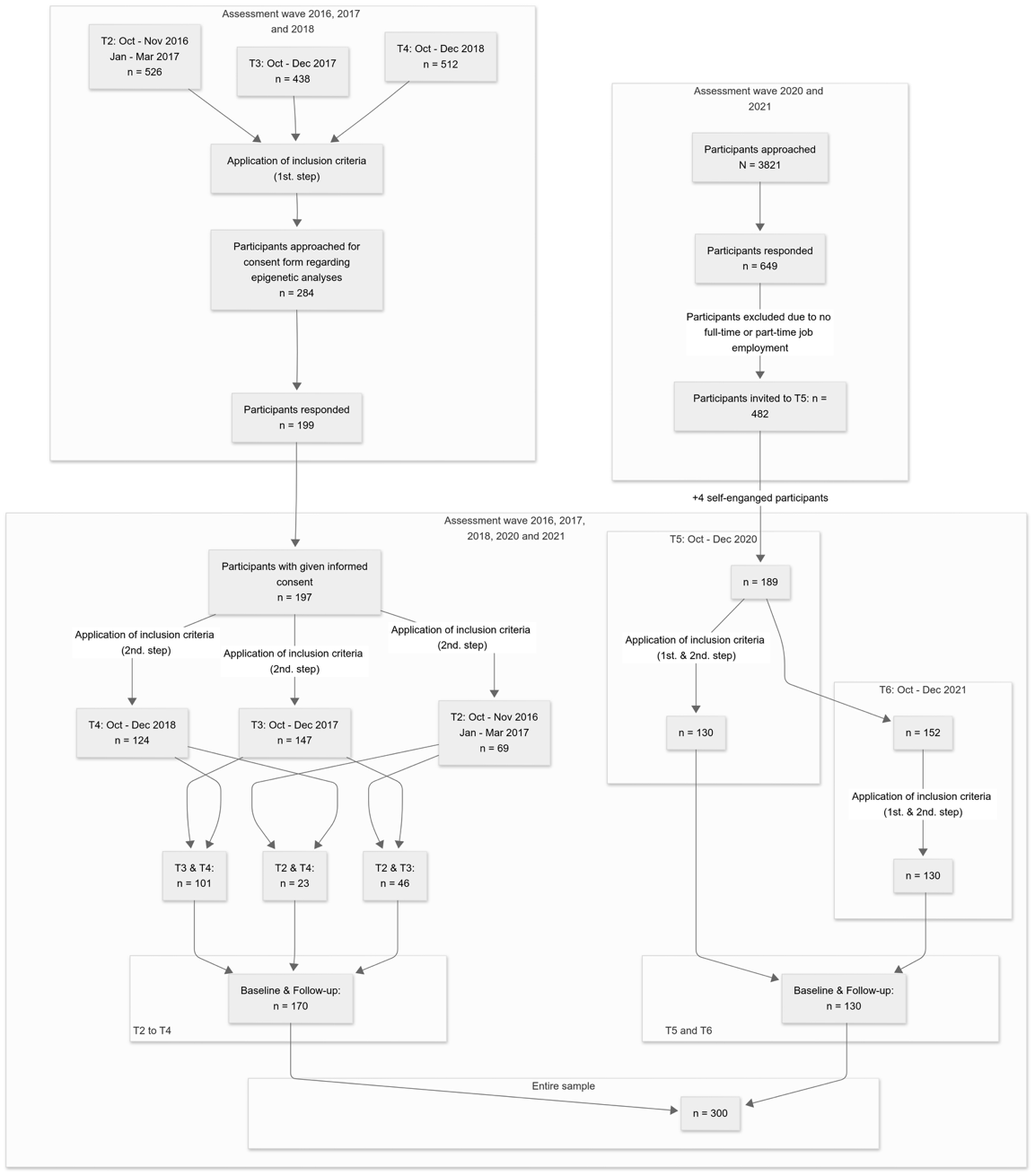


**Fig. S1** Flow Chart of the Sample Subjected to Epigenetic Analyses


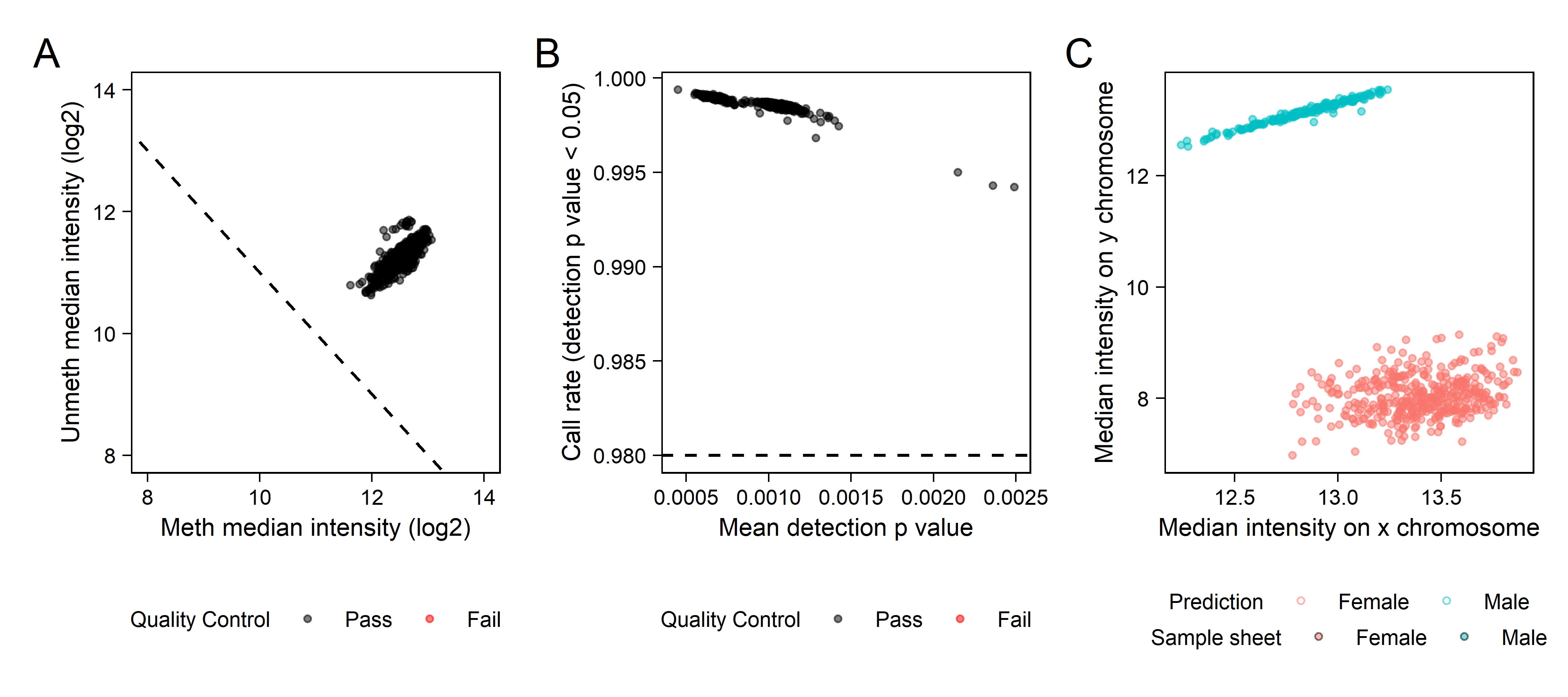


**Fig. S2 A** Median Intensity. **B** Call Rate. **C** Sex Check

**
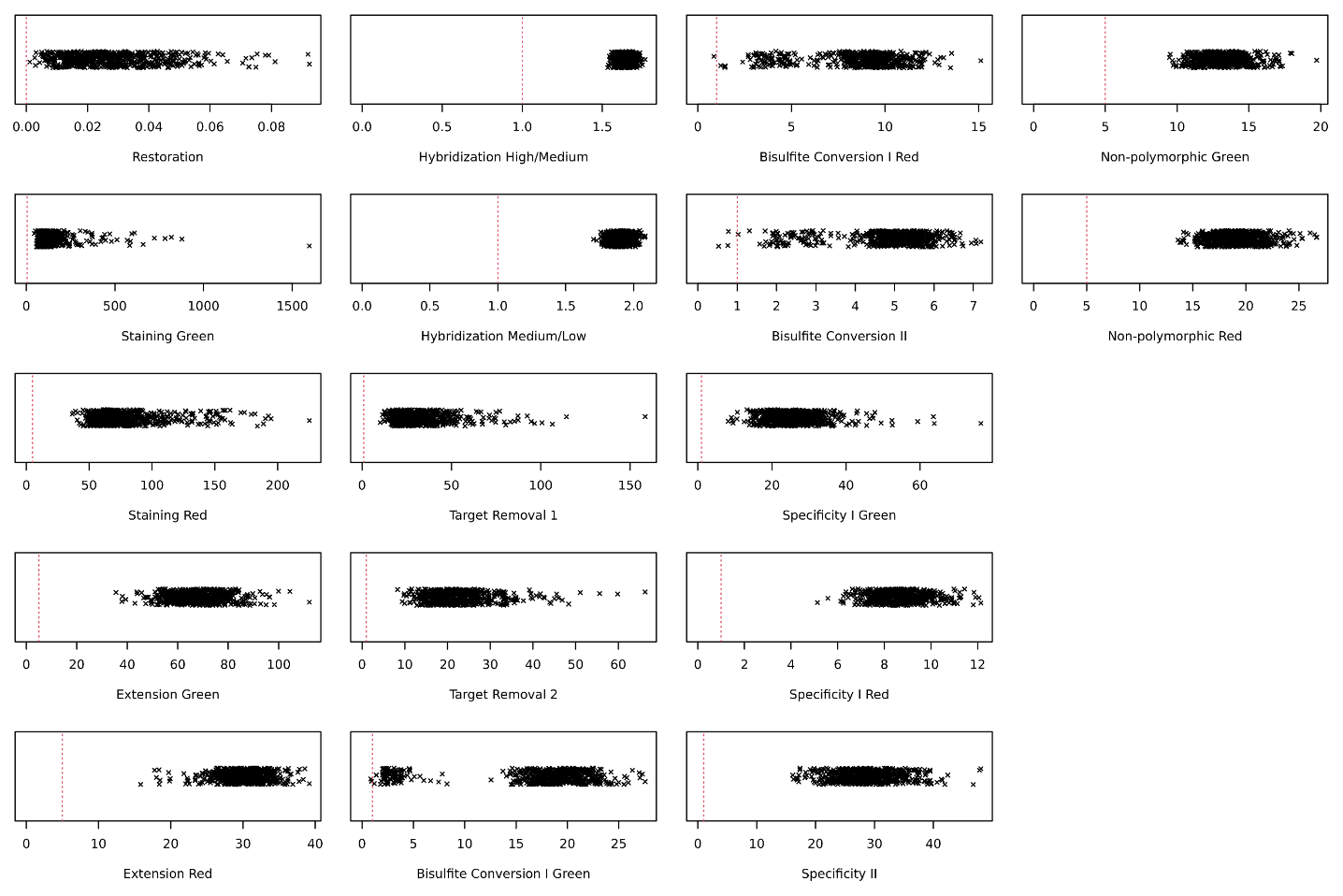
**

**Fig. S3** Illumina BeadArray Control Metrics


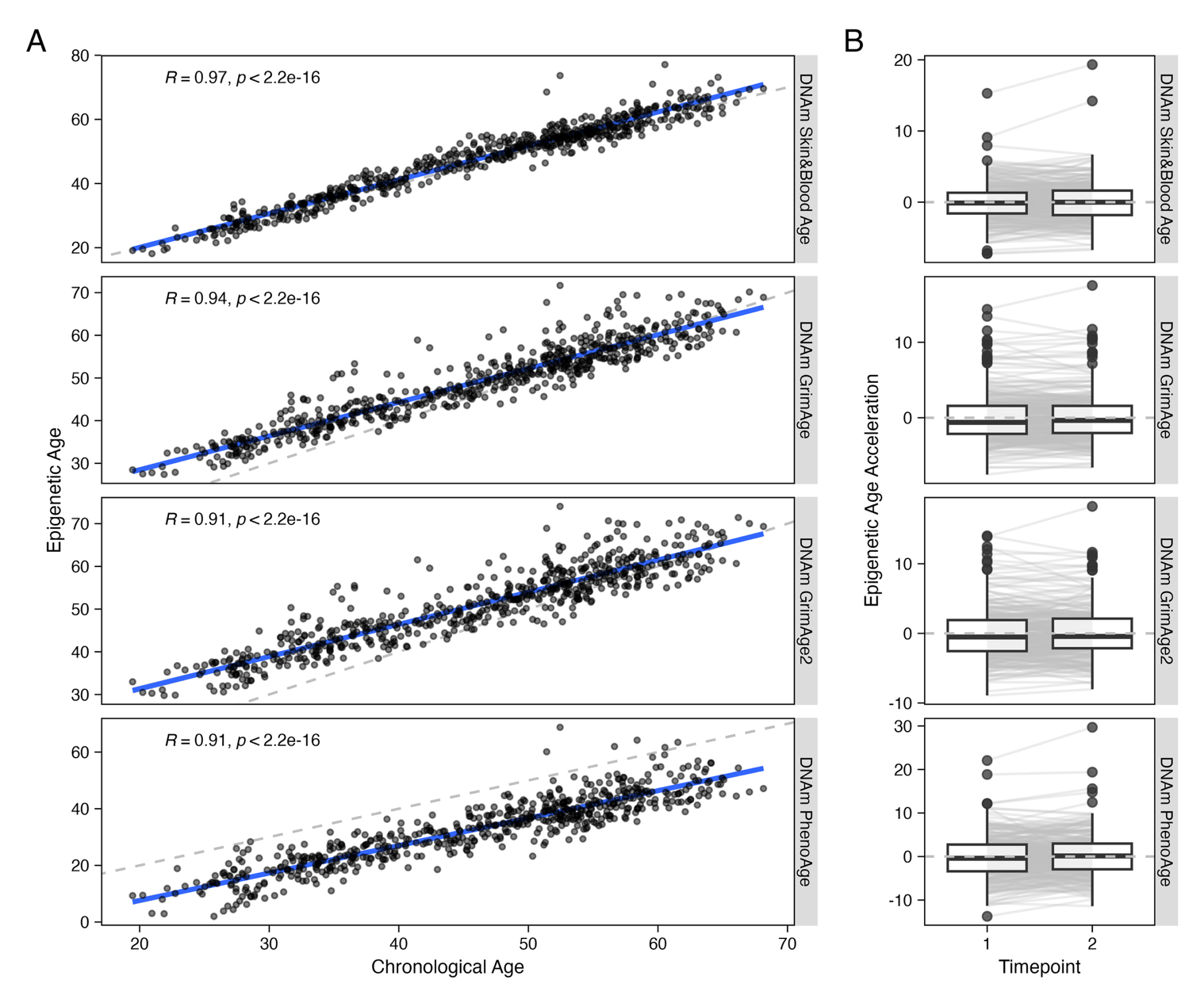


**Fig. S4** **A** Epigenetic Age Prediction. **B** Acceleration at Both Timepoints


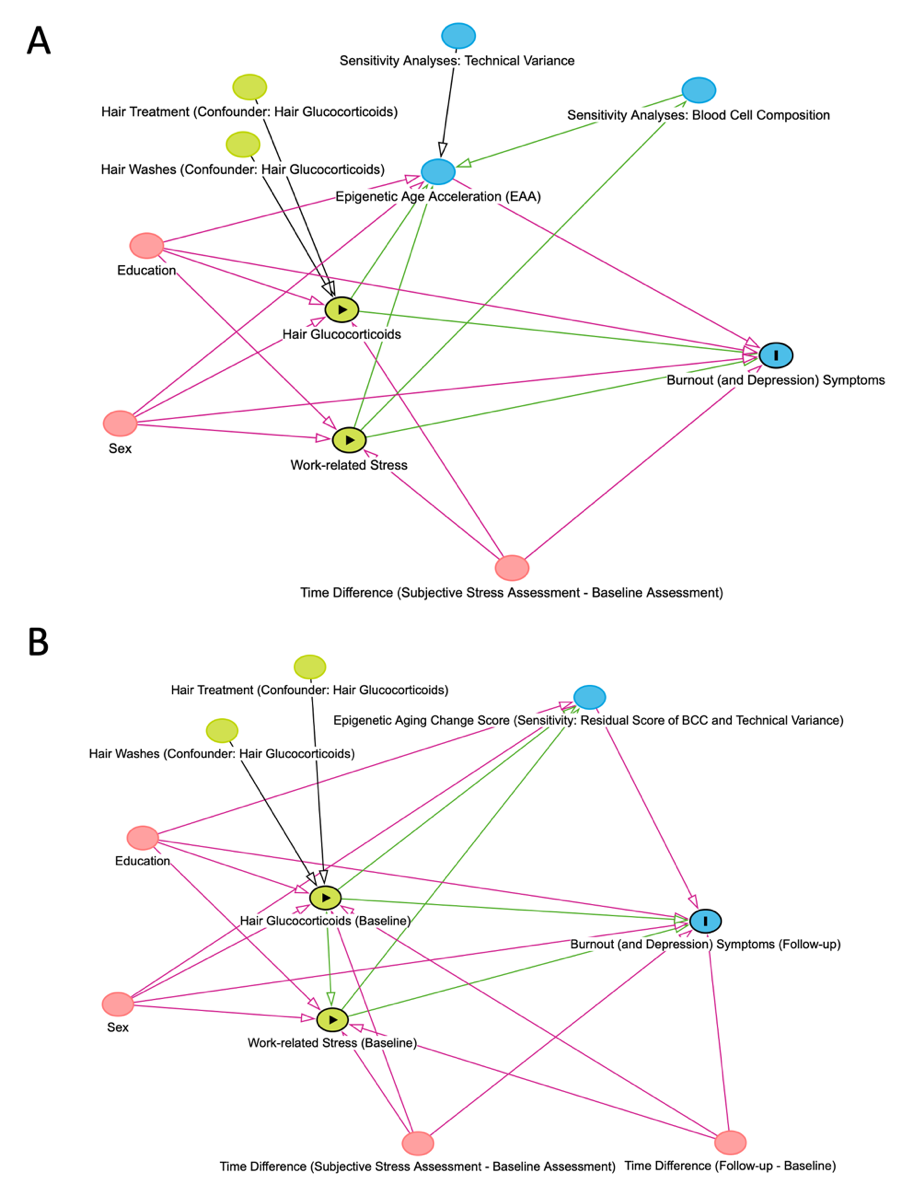


**Fig. S5** Directed Acyclic Graph of the underlying model. **A** Cross-sectional Mediation. **B** Longitudinal Mediation. BBC = blood cell composition (CD8, CD4, NK, B cells, Monocytes). Technical Variance = control probes PC1 and PC2.


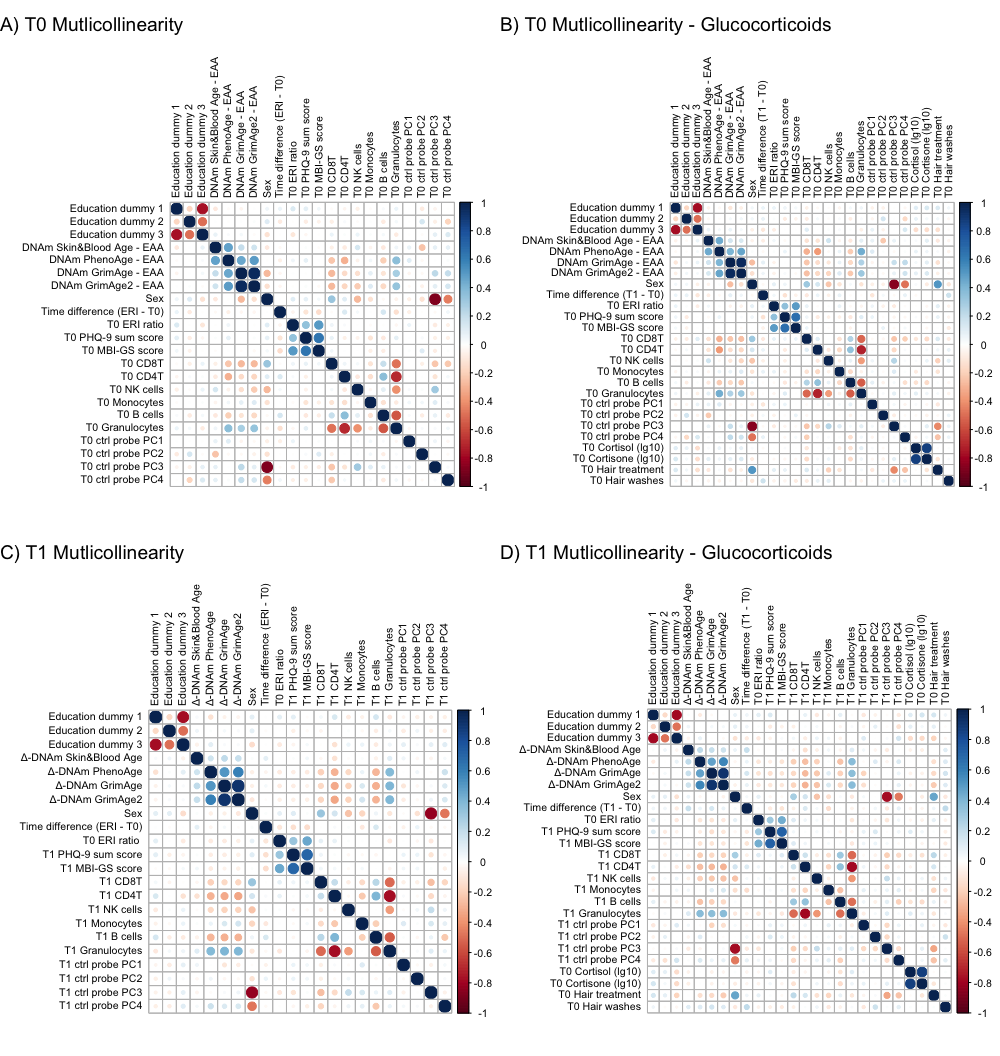


**Fig. S6** Multicollinearity of Baseline (T0) and Follow-up (T1) Variables With and Without Glucocorticoids. **A** T0 multicollinearity. **B** T0 multicollinearity with glucocorticoids. **C** T1 multicollinearity. **D** T1 multicollinearity with glucocorticoids

**Table S1** Mediating Role of EAA Regarding Work-related Stress or Hair Glucocorticoids and Depressive Symptoms

|  | **Outcome Regression Fit** | | | | | | **Mediation Paths, ß** | | | | | | | | **Indirect Effects** | | | |  |
| --- | --- | --- | --- | --- | --- | --- | --- | --- | --- | --- | --- | --- | --- | --- | --- | --- | --- | --- | --- |
|  | ***F ^HC4^*** | ***df*** | ***p*** | | **R^2^** | | ***a*** | | ***b*** | | ***c*** | | ***c‘*** | | **ß** | | **95% BootCI** | |  |
| **Model: ERI T0 🡪 Depressive symptoms T0**  **Confounders: sex, education dummy 1, education dummy 2, time difference ERI completion and baseline assessment** | | | | | | | | | | | | | | | | | | |  |
| DNAm Skin&Blood Age | 6.78 | 6, 239 | | <.001 | | .15 | | .02 | | .02 | | .33*** | | .33*** | | .0003 | | [-.01, .01] | |
| DNAm PhenoAge | 7.28 | 6, 239 | | <.001 | | .15 | | .06 | | .06 | | .33*** | | .33*** | | .004 | | [-.00, .02] | |
| DNAm GrimAge | 6.76 | 6, 239 | | <.001 | | .15 | | .04 | | .01 | | .33*** | | .33*** | | .0003 | | [-.01, .01] | |
| DNAm GrimAge2 | 6.88 | 6, 239 | | <.001 | | .15 | | .001 | | .03 | | .33*** | | .33*** | | .00005 | | [-.01, .01] | |
| **Model: HairF T0 🡪 Depressive symptoms T0**  **Confounders: sex, education dummy 1, education dummy 2, hair treatment, hair washes** | | | | | | | | | | | | | | | | | | |  |
| DNAm Skin&Blood Age | 0.72 | 7, 237 | | .657 | | .02 | | .03 | | -.03 | | .03 | | .03 | | -.0008 | | [-.01, .01] | |
| DNAm PhenoAge | 0.93 | 7, 237 | | .485 | | .02 | | .08 | | .06 | | .03 | | .02 | | .005 | | [-.01, .02] | |
| DNAm GrimAge | 0.83 | 7, 237 | | .564 | | .02 | | .05 | | .03 | | .03 | | .03 | | .001 | | [-.01, .01] | |
| DNAm GrimAge2 | 0.88 | 7, 237 | | .526 | | .02 | | .10 | | .04 | | .03 | | .02 | | .004 | | [-.01, .02] | |
| **Model: HairE T0 🡪 Depressive symptoms T0**  **Confounders: sex, education dummy 1, education dummy 2, hair treatment, hair washes** | | | | | | | | | | | | | | | | | | |  |
| DNAm Skin&Blood Age | 0.71 | 7, 244 | | .660 | | .02 | | .09 | | -.02 | | -.03 | | -.03 | | -.002 | | [-.02, .01] | |
| DNAm PhenoAge | 0.96 | 7, 244 | | .463 | | .02 | | .05 | | .08 | | -.03 | | -.04 | | .004 | | [-.01, .02] | |
| DNAm GrimAge | 0.85 | 7, 244 | | .546 | | .02 | | .08 | | .03 | | -.03 | | -.03 | | .003 | | [-.01, .02] | |
| DNAm GrimAge2 | 0.92 | 7, 244 | | .492 | | .02 | | .11 | | .05 | | -.03 | | -.04 | | .006 | | [-.01, .03] | |

*Note.* EAA: Epigenetic Age Acceleration. ERI: Effort-Reward Imbalance. DNAm: DNA Methylation. Reference education dummy 1: secondary school & middle school. Reference education dummy 2: vocational school. HairF = Hair cortisol concentration. HairE = hair cortisone concentration. Model sample size: T0 ERI - Depression: *n* = 246; T0 Cortisol: *n* = 245; T0 Cortisone: *n* = 252. *** *p* < .001.

**Table S2** Mediating Role of Delta_EA Regarding Work-related Stress or Hair Glucocorticoids T0 and Depressive Symptoms T1

|  | **Outcome Regression Fit** | | | | | | **Mediation Paths, ß** | | | | | | | | | | **Indirect Effects** | | | | |
| --- | --- | --- | --- | --- | --- | --- | --- | --- | --- | --- | --- | --- | --- | --- | --- | --- | --- | --- | --- | --- | --- |
|  | ***F ^HC4^*** | ***df*** | ***p*** | | **R^2^** | | ***a*** | | ***b*** | | ***c*** | | ***c‘*** | | **ß** | | | **95% BootCI** | |  |  |
| **Model: ERI T0 –> Depressive symptoms T1**  **Confounders: sex, education dummy 1, education dummy 2, time difference ERI completion and baseline assessment, time difference T1 - T0** | | | | | | | | | | | | | | | | | | | | | |
| Δ-DNAm Skin&Blood Age | 5.79 | 7, 240 | | <.001 | | .14 | | .03 | | .05 | | .32*** | | .32*** | | .001 | | | [-.01, .02] | |  |
| Δ-DNAm PhenoAge | 5.78 | 7, 240 | | <.001 | | .14 | | -.08 | | -.05 | | .32*** | | .31*** | | .005 | | | [-.01, .02] | |  |
| Δ-DNAm GrimAge | 5.66 | 7, 240 | | <.001 | | .14 | | -.10 | | .001 | | .32*** | | .32*** | | -.0001 | | | [-.01, .02] | |  |
| Δ-DNAm GrimAge2 | 5.70 | 7, 240 | | <.001 | | .14 | | -.08 | | -.001 | | .32*** | | .32*** | | .0007 | | | [-.01, .01] | |  |
| **Model: HairF T0 🡪 Depressive symptoms T1**  **Confounders: sex, education dummy 1, education dummy 2, hair treatment, hair washes, time difference T1 - T0** | | | | | | | | | | | | | | | | | | | | | |
| Δ-DNAm Skin&Blood Age | 1.07 | 8, 236 | | .382 | | .04 | | -.06 | | .01 | | .08 | | .08 | | -.0009 | | | [-.01, .01] | |  |
| Δ-DNAm PhenoAge | 1.02 | 8, 236 | | .423 | | .04 | | .01 | | -.05 | | .08 | | .08 | | -.0007 | | | [-.01, .01] | |  |
| Δ-DNAm GrimAge | 1.16 | 8, 236 | | .323 | | .04 | | -.07 | | -.08 | | .08 | | .07 | | .006 | | | [-.01, .03] | |  |
| Δ-DNAm GrimAge2 | 1.07 | 8, 236 | | .388 | | .04 | | -.06 | | -.06 | | .08 | | .07 | | .004 | | | [-.01, .02] | |  |
| **Model: HairE T0 🡪 Depressive symptoms T1**  **Confounders: sex, education dummy 1, education dummy 2, hair treatment, hair washes, time difference T1 - T0** | | | | | | | | | | | | | | | | | | | | | |
| Δ-DNAm Skin&Blood Age | 1.03 | 8, 243 | | .413 | | .03 | | -.10 | | .02 | | -.002 | | -.001 | | -.002 | | | [-.02, .01] | |  |
| Δ-DNAm PhenoAge | 1.01 | 8, 243 | | .431 | | .04 | | .01 | | -.05 | | -.002 | | -.002 | | -.0006 | | | [-.01, .01] | |  |
| Δ-DNAm GrimAge | 1.17 | 8, 243 | | .321 | | .04 | | -.09 | | -.08 | | -.002 | | -.01 | | .007 | | | [-.01, .03] | |  |
| Δ-DNAm GrimAge2 | 1.07 | 8, 243 | | .383 | | .04 | | -.07 | | -.06 | | -.002 | | -.006 | | .004 | | | [-.01, .03] | |  |

*Note.* Delta_EA: Change score of raw clock estimate at T1 - raw clock estimate at T0. ERI: Effort-Reward Imbalance. DNAm: DNA Methylation. Reference education dummy 1: secondary school & middle school. Reference education dummy 2: vocational school. Δ: Change in epigenetic aging between T0 and T1. HairF = Hair cortisol concentration. HairE = hair cortisone concentration. Model sample size: T0 ERI - Depression: *n* = 246; T0 Cortisol: *n* = 245; T0 Cortisone: *n* = 252. *** *p* < .001.

**Table S3** Sensitivity Analyses of Mediating Role of EAA Regarding Subjective or Biological Stress and Burnout or Depressive Symptoms

|  | **Outcome Regression Fit** | | | | **Mediation Paths, ß** | | | | **Indirect Effects** | |
| --- | --- | --- | --- | --- | --- | --- | --- | --- | --- | --- |
|  | ***F ^HC4^*** | ***df*** | ***p*** | **R^2^** | ***a*** | ***b*** | ***c*** | ***c‘*** | **ß** | **95% BootCI** |
| **Model: ERI T0 –> Burnout symptoms T0**  **Confounders: sex, education dummy 1, education dummy 2, time difference ERI completion and baseline assessment, ctrl-probe PC 1, ctrl-probe PC 2, CD8+, CD4+, NKC, monocytes, B cells** | | | | | | | | | | |
| DNAm Skin&Blood Age | 7.98 | 13, 234 | <.001 | .30 | .02 | .02 | .50*** | .50*** | .0004 | [-.01, .01] |
| DNAm PhenoAge | 8.01 | 13, 234 | <.001 | .30 | .07 | -.01 | .50*** | .50*** | -.0008 | [-.01, .01] |
| DNAm GrimAge | 7.79 | 13, 234 | <.001 | .30 | .06 | -.01 | .50*** | .50*** | -.0007 | [-.01, .01] |
| DNAm GrimAge2 | 7.97 | 13, 234 | <.001 | .30 | .02 | -.01 | .50*** | .50*** | -.0002 | [-.01, .01] |
| **Model: ERI T0 –> Depressive symptoms T0**  **Confounders: sex, education dummy 1, education dummy 2, time difference ERI completion and baseline assessment, ctrl-probe PC 2, ctrl-probe PC 1, CD8+, CD4+, NKC, monocytes, B cells)** | | | | | | | | | | |
| DNAm Skin&Blood Age | 3.54 | 13, 232 | <.001 | .17 | .01 | .03 | .33*** | .33*** | .0003 | [-.01, .01] |
| DNAm PhenoAge | 3.74 | 13, 232 | <.001 | .18 | .07 | .06 | .33*** | .33*** | .004 | [-.01, .02] |
| DNAm GrimAge | 3.49 | 13, 232 | <.001 | .17 | .06 | .0001 | .33*** | .33*** | .00001 | [-.01, .01] |
| DNAm GrimAge2 | 3.52 | 13, 232 | <.001 | .17 | .02 | .02 | .33*** | .33*** | .0006 | [-.01, .01] |
| **Model: HairF T0 🡪 Burnout symptoms T0**  **Confounders: sex, education dummy 1, education dummy 2, hair treatment, hair washes, ctrl-probe PC 1, ctrl-probe PC 2, CD8+, CD4+, NKC, monocytes, B cells** | | | | | | | | | | |
| DNAm Skin&Blood Age | 0.34 | 14, 230 | .988 | .03 | -.01 | .01 | -.001 | -.0009 | -.0001 | [-.01, .01] |
| DNAm PhenoAge | 0.34 | 14, 230 | .989 | .03 | .04 | -.005 | -.001 | -.0008 | -.0002 | [-.01, .01] |
| DNAm GrimAge | 0.34 | 14, 230 | .988 | .03 | .03 | .001 | -.001 | -.001 | .00004 | [-.01, .01] |
| DNAm GrimAge2 | 0.34 | 14, 230 | .987 | .03 | .07 | -.02 | -.001 | .0003 | -.001 | [-.02, .01] |
| **Model: HairF T0 🡪 Depressive symptoms T0**  **Confounders: sex, education dummy 1, education dummy 2, hair treatment, hair washes, ctrl-probe PC 1, ctrl-probe PC 2, CD8+, CD4+, NKC, monocytes, B cells** | | | | | | | | | | |
| DNAm Skin&Blood Age | 1.20 | 14, 230 | .275 | .05 | -.01 | -.04 | .01 | .01 | .0005 | [-.01, .02] |
| DNAm PhenoAge | 1.31 | 14, 230 | .201 | .05 | .04 | .04 | .01 | .01 | .002 | [-.01, .02] |
| DNAm GrimAge | 1.25 | 14, 230 | .238 | .05 | .03 | .02 | .01 | .01 | .0005 | [-.01, .01] |
| DNAm GrimAge2 | 1.29 | 14, 230 | .215 | .05 | .07 | .02 | .01 | .01 | .002 | [-.01, .02] |
| **Model: HairE T0 🡪 Burnout symptoms T0**  **Confounders: sex, education dummy 1, education dummy 2, hair treatment, hair washes, ctrl-probe PC 1, ctrl-probe PC 2, CD8+, CD4+, NKC, monocytes, B cells** | | | | | | | | | | |
| DNAm Skin&Blood Age | 0.32 | 14, 237 | .992 | .03 | .04 | -.004 | -.02 | -.02 | -.0002 | [-.01, .01] |
| DNAm PhenoAge | 0.32 | 14, 237 | .992 | .03 | .03 | -.01 | -.02 | -.02 | -.0003 | [-.01, .01] |
| DNAm GrimAge | 0.32 | 14, 237 | .991 | .03 | .05 | .004 | -.02 | -.02 | .0002 | [-.01, .01] |
| DNAm GrimAge2 | 0.32 | 14, 237 | .991 | .03 | .08 | -.02 | -.02 | -.02 | -.001 | [-.02, .02] |
| **Model: HairE T0 🡪 Depressive symptoms T0**  **Confounders: sex, education dummy 1, education dummy 2, hair treatment, hair washes, ctrl-probe PC 1, ctrl-probe PC 2, CD8+, CD4+, NKC, monocytes, B cells** | | | | | | | | | | |
| DNAm Skin&Blood Age | 1.18 | 14, 237 | .294 | .05 | .04 | -.04 | -.03 | -.03 | -.002 | [-.02, .01] |
| DNAm PhenoAge | 1.28 | 14, 237 | .221 | .05 | .03 | .05 | -.03 | -.03 | .001 | [-.01, .02] |
| DNAm GrimAge | 1.22 | 14, 237 | .265 | .05 | .05 | .02 | -.03 | -.03 | .001 | [-.01, .01] |
| DNAm GrimAge2 | 1.26 | 14, 237 | .232 | .05 | .08 | .03 | -.03 | -.04 | .003 | [-.01, .02] |

*Note.* EAA: Epigenetic Age Acceleration. ERI: Effort-Reward Imbalance. DNAm: DNA Methylation. Reference education dummy 1: secondary school & middle school. Reference education dummy 2: vocational school. HairF = Hair cortisol concentration. HairE = hair cortisone concentration. Ctrl-probe PC1: Illumina control probe principal component 1. Ctrl-probe PC2: Illumina control probe principal component 2. NKC: Natural killer cells. Model sample size: T0 ERI – Burnout: *n* = 248; T0 ERI – Depression: *n* = 246; T0 Cortisol: *n* = 245; T0 Cortisone: *n* = 252. *** *p* < .001.

**Table S4** Sensitivity Analyses of Mediating Role of Delta_EA Regarding Subjective or Biological Stress T0 and Burnout or Depressive Symptoms T1

|  | **Outcome Regression Fit** | | | | | | | | **Mediation Paths, ß** | | | | | | | | | | | | **Indirect Effects** | | | | | |  |  |
| --- | --- | --- | --- | --- | --- | --- | --- | --- | --- | --- | --- | --- | --- | --- | --- | --- | --- | --- | --- | --- | --- | --- | --- | --- | --- | --- | --- | --- |
|  | ***F ^HC4^*** | ***df*** | ***p*** | | | **R^2^** | | | ***a*** | | | ***b*** | | | ***c*** | | | ***c‘*** | | | **ß** | | | **95% BootCI** | | |  |  |
| **Model: ERI T0 –> Burnout symptoms T1**  **Confounders: sex, education dummy 1, education dummy 2, time difference ERI completion and baseline assessment, time difference T1 - T0** | | | | | | | | | | | | | | | | | | | | | | | | | | |  |  |
| Δ-DNAm Skin&Blood Age | 12.99 | 7, 240 | | <.001 | | | .24 | | | .01 | | | .11*^a^ | | | .47*** | | | .47*** | | | .001 | | | [-.01, .02} | | |  |
| Δ-DNAm PhenoAge | 11.04 | 7, 240 | | <.001 | | | .23 | | | -.06 | | | -.01 | | | .47*** | | | .47*** | | | .0008 | | | [-.01, .01] | | |  |
| Δ-DNAm GrimAge | 13.14 | 7, 240 | | <.001 | | | .23 | | | -.06 | | | .09 | | | .47*** | | | .47*** | | | -.005 | | | [-.02, .01] | | |  |
| Δ-DNAm GrimAge2 | 12.50 | 7, 240 | | <.001 | | | .23 | | | -.05 | | | .05 | | | .47*** | | | .47*** | | | -.002 | | | [-.01, .01] | | |  |
| **Model: ERI T0 –> Depressive symptoms T1**  **Confounders: sex, education dummy 1, education dummy 2, time difference ERI completion and baseline assessment, time difference T1 - T0,** | | | | | | | | | | | | | | | | | | | | | | | | | | |  |  |
| Δ-DNAm Skin&Blood Age | 6.54 | 7, 240 | | <.001 | | | .15 | | | .01 | | | .10 | | | .32*** | | | .32*** | | | .001 | | | [-.01, .02] | | |  |
| Δ-DNAm PhenoAge | 5.78 | 7, 240 | | <.01 | | | .14 | | | -.06 | | | -.01 | | | .32*** | | | .32*** | | | .0005 | | | [-.01, .01] | | |  |
| Δ-DNAm GrimAge | 6.15 | 7, 240 | | <.001 | | | .14 | | | -.06 | | | .06 | | | .32*** | | | .33*** | | | -.004 | | | [-.02, .01] | | |  |
| Δ-DNAm GrimAge2 | 6.06 | 7, 240 | | <.001 | | | .14 | | | -.05 | | | .05 | | | .32*** | | | .32*** | | | -.002 | | | [-.01, .01] | | |  |
| **Model: HairF T0 🡪 Burnout symptoms T1**  **Confounders: sex, education dummy 1, education dummy 2, hair treatment, hair washes, time difference T1 - T0** | | | | | | | | | | | | | | | | | | | | | | | | | | |  |  |
| Δ-DNAm Skin&Blood Age | 0.15 | 8, 236 | | .996 | | | .01 | | | .05 | | | .04 | | | .01 | | | .004 | | | .002 | | | [-.01, .02] | | |  |
| Δ-DNAm PhenoAge | 0.21 | 8, 236 | | .989 | | | .01 | | | .07 | | | -.05 | | | .01 | | | .01 | | | -.004 | | | [-.02, .01] | | |  |
| Δ-DNAm GrimAge | 0.15 | 8, 236 | | .997 | | | .01 | | | .04 | | | -.01 | | | .006 | | | .006 | | | -.0004 | | | [-.01, .01] | | |  |
| Δ-DNAm GrimAge2 | 0.16 | 8, 236 | | .996 | | | .01 | | | .03 | | | -.02 | | | .006 | | | .006 | | | -.0006 | | | [-.01, .01] | | |  |
| **Model: HairF T0 🡪 Depressive symptoms T1**  **Confounders: sex, education dummy 1, education dummy 2, hair treatment, hair washes, time difference T1 - T0** | | | | | | | | | | | | | | | | | | | | | | | | | | |  |  |
| Δ-DNAm Skin&Blood Age | 1.04 | 8, 236 | | | .401 | | | .04 | | | .05 | | | .01 | | | .08 | | | .08 | | | .0005 | | | [-.01, .02] | | |
| Δ-DNAm PhenoAge | 1.06 | 8, 236 | | | .393 | | | .04 | | | .07 | | | -.04 | | | .08 | | | .08 | | | -.003 | | | [-.02, .01] | | |
| Δ-DNAm GrimAge | 1.41 | 8, 236 | | | .337 | | | .04 | | | .04 | | | -.04 | | | .08 | | | .08 | | | -.002 | | | [-.02, .01] | | |
| Δ-DNAm GrimAge2 | 1.08 | 8, 236 | | | .375 | | | .04 | | | .03 | | | -.03 | | | .08 | | | .08 | | | -.0008 | | | [-.01, .01] | | |
| **Model: HairE T0 🡪 Burnout symptoms T1**  **Confounders: sex, education dummy 1, education dummy 2, hair treatment, hair washes, time difference T1 - T0** | | | | | | | | | | | | | | | | | | | | | | | | | | |  |  |
| Δ-DNAm Skin&Blood Age | 0.20 | 8, 243 | | | .990 | | | .01 | | | .06 | | | .04 | | | -.03 | | | -.04 | | | .002 | | | [-.01, .02] | | |
| Δ-DNAm PhenoAge | 0.26 | 8, 243 | | | .978 | | | .02 | | | .07 | | | -.05 | | | -.03 | | | -.03 | | | -.003 | | | [-.02, .01] | | |
| Δ-DNAm GrimAge | 0.20 | 8, 243 | | | .991 | | | .01 | | | .03 | | | -.01 | | | -.03 | | | -.03 | | | -.0003 | | | [-.01, .01] | | |
| Δ-DNAm GrimAge2 | 0.22 | 8, 243 | | | .987 | | | .01 | | | .03 | | | -.02 | | | -.03 | | | -.03 | | | -.0007 | | | [-.01, .01] | | |
| **Model: HairE T0 🡪 Depressive symptoms T1**  **Confounders: sex, education dummy 1, education dummy 2, hair treatment, hair washes, time difference T1 - T0** | | | | | | | | | | | | | | | | | | | | | | | | | | |  |  |
| Δ-DNAm Skin&Blood Age | 1.02 | 8, 243 | | | .424 | | | .03 | | | .06 | | | .001 | | | -.002 | | | -.002 | | | .00004 | | | [-.01, .02] | | |
| Δ-DNAm PhenoAge | 1.03 | 8, 243 | | | .411 | | | .04 | | | .07 | | | -.05 | | | -.002 | | | .001 | | | -.003 | | | [-.02, .01] | | |
| Δ-DNAm GrimAge | 1.34 | 8, 243 | | | .341 | | | .04 | | | .03 | | | -.05 | | | -.002 | | | -.0004 | | | -.002 | | | [-.02, .01] | | |
| Δ-DNAm GrimAge2 | 1.09 | 8, 243 | | | .369 | | | .04 | | | .03 | | | -.05 | | | -.002 | | | -.0008 | | | -.002 | | | [-.02, .01] | | |

*Note.* Delta_EA: Change score of raw clock estimate at T1 - raw clock estimate at T0. ERI: Effort-Reward Imbalance. DNAm: DNA Methylation. Reference education dummy 1: secondary school & middle school. Reference education dummy 2: vocational school. Δ: Change in epigenetic aging between T0 and T1. HairF = Hair cortisol concentration. HairE = hair cortisone concentration. DNAm change scores are defined as the residual score of cell mixture distribution (CD8+, CD4+, NKC, monocytes, B cells) and two Illumina control probe principal components (ctrl-probe PC 1, ctrl-probe PC 2). Ctrl-probe PC1: Illumina control probe principal component 1. Ctrl-probe PC2: Illumina control probe principal component 2. NKC: Natural killer cells. Model sample size: T0 ERI – Burnout: *n* = 248; T0 ERI – Depression: *n* = 246; T0 Cortisol: *n* = 245; T0 Cortisone: *n* = 252. ^a^ 95% BCI[.00, .09]. * *p* < .05. *** *p* < .001.
